# Does *Bacillus anthracis* Lethal Toxin Directly Depress Myocardial Function? A Review of Clinical Cases and Preclinical Studies

**DOI:** 10.3390/toxins7124891

**Published:** 2015-12-12

**Authors:** Dante A. Suffredini, Hanish Sampath-Kumar, Yan Li, Lernik Ohanjanian, Kenneth E. Remy, Xizhong Cui, Peter Q. Eichacker

**Affiliations:** 1 Critical Care Medicine Department, Clinical Center, National Institutes of Health, Bethesda, MD 20892, USA; hanishsampathkumar@gmail.com (H.S.-K.); yli3@cc.nih.gov (Y.L.); lernik.ohanjanian@nih.gov (L.O.); CXizhong@cc.nih.gov (X.C.); PEichacker@cc.nih.gov (P.Q.E.); 2 Division of Critical Care Medicine, Department of Pediatrics, Washington University School of Medicine, St. Louis, MO 63110, USA; Remy_K@kids.wustl.edu

**Keywords:** *Bacillus anthracis*, anthrax, lethal and edema toxins, cardiovascular dysfunction, shock, treatment

## Abstract

The US outbreak of *B.anthracis* infection in 2001 and subsequent cases in the US and Europe demonstrate that anthrax is a continuing risk for the developed world. While several bacterial components contribute to the pathogenesis of *B. anthracis*, production of lethal toxin (LT) is strongly associated with the development of hypotension and lethality. However, the mechanisms underlying the cardiovascular instability LT produces are unclear. Some evidence suggests that LT causes shock by impairing the peripheral vasculature, effects consistent with the substantial extravasation of fluid in patients dying with *B. anthracis*. Other data suggests that LT directly depresses myocardial function. However a clinical correlate for this latter possibility is less evident since functional studies and post-mortem examination in patients demonstrate absent or minimal cardiac changes. The purposes of this review were to first present clinical studies of cardiac functional and histologic pathology with *B. anthracis* infection and to then examine *in vivo*, *in vitro*, and *ex vivo* preclinical studies of LT’s myocardial effects. Together, these data suggest that it is unclear whether that LT directly depresses cardiac function. This question is important for the clinical management and development of new therapies for anthrax and efforts should continue to be made to answer it.

## 1. Introduction

The development of shock in patients with *Bacillus anthracis* (*B. anthracis* or anthrax*)* infection appears associated with a particularly poor prognosis. In contrast to mortality rates of 40% to 50% with shock complicating other types of bacterial infection, in recent anthrax outbreaks in the US and Europe, mortality rates in patients with shock have been greater than 75% [1,2,3,4]. *B. anthracis* produces two toxins, lethal and edema toxins (LT and ET respectively), as well as other components (e.g., non-toxin proteases and cell wall components) which may contribute to its pathogenesis [5]. Of these components however, production of LT has been most strongly associated with the development of hypotension and subsequent lethality. Despite increasing insights into LT’s intracellular effects, the mechanisms underlying the cardiovascular instability it produces are unclear.

On the other hand, some experimental data have also suggested that LT produces shock by impairing the peripheral vasculature via either disruption of endothelial barrier or vascular smooth muscle function [6,7,8,9,10]. Such effects would be very consistent with the substantial extravasation of fluid and resistance to conventional hemodynamic support that has been noted to occur in patients and animals dying with *B. anthracis* infection [1,2,11,12]. On the other hand, some experimental data has also suggested that LT produces shock by directly depressing myocardial systolic and/or diastolic function [13,14,15,16,17]. However a clinical correlate to this possibility is less evident. Although data regarding cardiac function in patients with *B. anthracis* are limited, there are several striking examples of patients dying with anthrax associated shock but in whom echocardiographic and even pulmonary arterial catheter measures of myocardial function close to the time of death were reported to be normal [18,19]. Also, while LT has been reported to depress myocardial function in animal models, these observations have sometimes been based on calculated reductions in cardiac output (CO) or left ventricular ejection fraction (LVEF) measured with echocardiography [10,13,20]. These studies can be difficult to interpret since the influence of preload or afterload changes on heart volumes, CO or LVEF have typically not been accounted for.

Given the likely important contribution LT makes to the development of shock during *B. anthracis* infection, a clearer understanding of whether it does in fact directly depress myocardial function has important clinical and research implications. First, it would emphasize the need for practitioners to directly monitor myocardial function in patients with invasive anthrax infection and cardiovascular instability and to consider the early use of vasoactive agents with strong inotropic effects. Second, it would highlight the need for research directed at identifying mechanisms underlying LT induced myocardial defects and the agents best able to correct those. After a short description of LT and its intracellular actions, the purpose of the present review is to first examine whether clinical data implicates cardiac dysfunction in the pathogenesis of *B. anthracis* infection and to then summarize *in vivo*, *in vitro*, and *ex vivo* preclinical data investigating LT’s potential effects on myocardial function.

## 2. Lethal Toxin and Its Intracellular Actions

Lethal toxin is a binary type toxin comprised of protective antigen (PA), the component necessary for toxin uptake by host cells, and lethal factor (LF), the toxic component [9,21]. During infection, both PA and LF are released into the circulation. A PA protomer (PA_83_) binds to one of at least two receptors on host cells; tumor endothelial marker-8 (TEM-8) or capillary morphogenesis gene-2 (CMG2), also termed anthrax toxin receptors 1 or 2 (ANTR-1 or ANTR-2), respectively. Both receptors are widely distributed in host tissues including the heart and blood vessels although some data suggests that CMG2 binding plays a predominant role in LT’s effects during infection [9]. The PA_83_ protomer then undergoes furin cleavage, and the remaining bound monomer (PA_63_) forms heptamers or octamers that up to three or four LF molecules (or edema factor [EF] molecules, the toxic moiety of ET) can bind to. This complex then undergoes endocytosis and as the endosomal pH decreases, the LF (and EF) molecules are released into the host cell’s cytosol where they can act.

Lethal factor is a metalloprotease which has two primary actions: it cleaves mitogen activated protein kinase kinase (MAPKK) pathways 1 to 4, 6 and 7, thus disrupting key host stress kinase pathways (ERK 1 and 2, p38 and JNK 1) and; it activates the host inflammasome NLRP3, resulting in upregulation of caspase-1 and the production of IL-1 and IL-12 [22,23]. Evidence suggests that both of these actions have the potential to produce relatively acute and direct myocardial injury and dysfunction [24,25].

## 3. Effects of *B. Anthracis* Infection on the Heart in Clinical Studies

As noted above, studies investigating myocardial function in patients with *B. anthracis* are very limited. Furthermore, any abnormalities that have been reported cannot be attributed to LT alone given the multiple other bacterial components associated with this bacterium that could alter the heart [5]. Other types of gram-positive bacterial infection have been associated with myocardial depression in the absence of LT [26]. It is still worthwhile though to consider what is known about the myocardial effects of *B. anthracis* infection in patients as a basis for considering what LT might contribute to. In this context, clinical data are primarily comprised of histopathology findings from patients dying with anthrax infection and of functional studies available in a small number of isolated case reports.

The largest group of anthrax cases for which pathological data are available comes from the outbreak of inhalational infection in Sverdlovsk, Russia in 1979, which affected at least 94 people, 64 of whom died [11,12]. This outbreak resulted from the inadvertent release of anthrax spores from a bioweapons factory. Quantitative histological examination of 41 autopsy cases from this outbreak was noteworthy for extensive bacterial involvement of the mediastinum and mediastinal lymph nodes with associated tissue edema, hemorrhage, fibrin deposition, and vasculitis, and with spread of these changes to the intestines and central nervous system (CNS) in some cases. Large pleural effusions and retrograde lymphatic spread of bacteria producing pneumonia were observed to have likely resulted in respiratory compromise. Investigators speculated that much of the changes noted were related to initial foci of bacteria releasing LT and ET, which in turn produced loss of endothelial integrity, edema formation, hemorrhage, and subsequently apoptotic cell death. Despite extensive changes in several organs though, examination of heart tissue in 25 patients demonstrated bacteria in only nine specimens. Furthermore, there were no specific cardiac microscopic findings noted, with exception of a few cases showing myocyte hypereosinophilia and rare focal-contraction band necrosis attributed to agonal hypotension and hypoxemia.

Another group of clinical autopsies (five) or tissue samples (three lung biopsies) available for review comes from the outbreak of inhalational anthrax affecting 11 patients in the United States in 2001 [1,27]. This outbreak resulted from the dissemination of anthrax contaminated letters. While tissue examination was reported individually from several of these cases, a systematic analysis was also performed of all eight cases. This analysis reported that the major pathological and histopathological findings came from immunohistochemistry (IHC) staining which showed *B. anthracis* cell wall and capsule fragments in mediastinal lymph nodes, soft tissue, and pleura and in the blood vessels or sinusoids of lung, liver, spleen, and intestines. Notably, findings related to the heart in patients with autopsies were not specifically recorded, other than that rare bacteria were present in coronary vessels. Individual reports from these five cases concentrated on mediastinal (lymph nodes and soft tissue), lung, intestinal and CNS tissue examination and none reported findings specifically regarding the heart or myocardium [28,29,30,31].

In three autopsy cases reported from an outbreak of *B. anthracis* among wool workers, there was no mention of abnormal myocardial changes on microscopic examination in two, while moderate sub-endocardial hemorrhage was noted in one [32]. In a review of 33 autopsy cases, while microscopic changes were described in the lung, gastrointestinal tract, lymph nodes, spleen, liver, and kidney, there was no report of abnormal findings on heart examination [33].

Thus in these reports, there appeared to be limited histologic evidence of myocardial injury in patients dying with *B. anthracis* infection. However, this does not exclude the possibility that electron microscopy or other histologic methods might not have shown evidence of myocardial involvement.

Functional cardiac studies from patients with *B. anthracis* infection are very limited. Over the past 15 years there have been approximately 80 cases of inhalational, gastrointestinal, or injectional anthrax (*i.e.*, the three forms of infection associated with high mortality rates) reported to have occurred in the US or Europe [1,2,34,35,36,37,38]. Of these, 48 appear to have actually been noted in the literature either in individual case reports or case series. Among these reports, descriptions of functional cardiac studies have been provided in only 11 patients. The most detailed study was of a 61 year old woman, who presented during the 2001 *B. anthracis* outbreak with respiratory distress with bilateral pleural effusions, peri-hilar infiltrates and a widened mediastinum on chest radiograph [18]. Echocardiogram at presentation showed normal left ventricular function and wall motion, slight aortic regurgitation, a small pericardial effusion and an aneurysm of the ascending aorta. EKG showed sinus tachycardia but was otherwise normal and a troponin level was negative. Although treated initially with furosemide for presumed heart failure, the patient’s respiratory and hemodynamic status rapidly worsened and she was intubated and mechanically ventilated. A pulmonary artery catheter was placed which showed a right atrial pressure of 4 mmHg (reference range 0–6 mmHg), right ventricular pressure of 17/5 mmHg (reference range 20–30/5–15 mmHg) and a pulmonary artery pressure of 20/10 mmHg (reference range of 20–30/5–15 mmHg). Pulmonary artery occlusion pressure (PAOP) was unobtainable and cardiac output was not reported. The patient then received volume resuscitation for hemodynamic instability and vasopressors were ultimately required. After initial testing, differential diagnosis included dissecting aortic aneurysm, severe community acquired pneumonia, vasculitis, or anthrax, but not heart failure. Blood cultures from admission grew *B. anthracis*. Echocardiography on day 2 showed an increase in the size of the pericardial effusion. The patient’s respiratory status continued to worsen and a repeat echocardiogram on day 3 showed further increase in the pericardial effusion and mild to moderate right atrial and ventricular collapse during diastole. Cardiac index was 2.6 L/min/m^2^ (reference range 2.4 to 4.0 L/min/m^2^), systemic vascular resistance was 1131 dynes·s·m^2^/cm^5^ (reference range 900–1400 dynes·s·m^2^/cm^5^) and a pulmonary capillary wedge pressure of 13 mmHg (reference range 6–12 mmHg) with no evidence of equalization of pressures. A follow-up echocardiogram 5 h later showed persistent collapse of the right atrium and ventricle concerning for tamponade. An attempted pericardiocentesis was unsuccessful and the patient expired. Autopsy showed a hemorrhagic pericardial effusion.

Other clinical reports of cardiac assessment in anthrax patients provide less information. Echocardiography was performed close to the time of admission in a 44 year old male patient subsequently found to have inhalational anthrax related to infected animal hides and who presented with two to three days of fever, respiratory symptoms, and evidence of pneumonia with a loculated pleural effusion [35]. Echocardiography showed a slightly dilated and mild to moderately hypertrophied left ventricle with a mildly hypokinetic apex, contractility at the lower limits of normal for the rest of the ventricle and a small pericardial effusion (personal communication with author). This patient developed progressive respiratory distress related to his pulmonary disease and hemodynamic compromise requiring mechanical ventilation and circulatory support but additional echocardiographic findings were not reported. In a 61 year old man with inhalational anthrax of unclear origin who presented with progressive respiratory failure requiring mechanical ventilation but who remained hemodynamically stable, an echocardiogram on day six of hospitalization showed left ventricular hypertrophy, mildly decreased LVEF (40%), no wall motion abnormality, an estimated pulmonary artery systolic pressure of 17 mmHg, probable decreased right ventricular function and no pericardial effusion (personal communication with author) [38]. A cardiac troponin I (cTnI) level close to the time of the echocardiogram was 0.025 ng/mL (reference value <0.034 ng/mL). The patient survived but no follow up echocardiogram was performed. A 55 year old man with severe injectional anthrax involving his thigh and peritoneum developed fulminant septic shock that progressed to cardiac arrest and was resuscitated after 12 min of CPR. A transesophageal echocardiogram performed on day 2 of hospitalization, after his cardiac arrest and while on high dose vasopressors and with systemic acidosis, was reported as normal [19]. This patient died on day 4 of hospitalization with progressive multi-organ failure but no subsequent echocardiogram was provided. A 24 year old woman presenting with nine days of progressive fatigue, fever, abdominal pain, and shock was later found to have gastrointestinal anthrax. An echocardiogram completed at admission was reportedly normal [37]. Finally, a survey of physicians that cared for 27 patients with injectional anthrax investigated multiple clinical end points pertaining to physical and laboratory findings, management and outcome. In this study, four patients reportedly had echocardiography while one patient had lithium dilution measures and another pulse contour cardiac output analysis [2]. Of these patients, three were noted to have normal echocardiograms, while the remaining three, all non-survivors, were reported to have abnormal findings with their respective tests, but actual data were not recorded. Other case reports of clinical anthrax have not included any functional or histopathologic cardiac data [27,39,40,41,42,43,44].

Table 1 summarizes cardiac findings from the clinical studies. Overall, these studies do not provide a clear picture as to whether *B. anthracis* infection is or is not associated with myocardial depression. It is unknown whether the limited data available regarding functional cardiac studies in clinical reports is the result of incomplete testing or because the results of such testing were unremarkable and not thought relevant for reporting.

**Table 1:** Summary of cardiac findings or the absence of any reported findings from clinical anthrax studies.

Publication	Number of Patients	Type of Anthrax Infection	Clinical or Histopathological Cardiac Findings
Albrink *et al.* 1960 [32]	3	Inhalational	Moderate subendocardial hemorrhage into myocardium of left ventricle noted at autopsy
Abramova *et al.* 1993 [11] Grinberg *et al.* 2001 [12]	42	Inhalational	No specific cardiac histopathological findings were noted. However, occasional cases showed myocyte hypereosinophilia and rare focal contraction band necrosis attributed to agonal hypotension and hypoxemia
Mayer *et al.* 2001 [44]	2	Inhalational	No functional cardiac assessment completed
Borio *et al.* 2001 [29]	2	Inhalational	ECG showing atrial fibrillation, no other specific findings noted
Jernigan *et al.* 2001 [1]	10 *	Inhalational	ECG showing atrial fibrillation noted in one of the four patients not reported as isolated cases
Bush *et al.* 2001 [28]	1	Inhalational	No gross cardiac abnormalities on autopsy found
Barakat *et al.* 2002 [31]	1	Inhalational	No functional cardiac assessment completed
Mina *et al.* 2002 [18]	1	Inhalational	Echocardiogram showed normal LV function at presentation with small pericardial effusion that enlarged and progressed to tamponade. Pulmonary artery catheterization also completed (see Section 3)
Guarner *et al.* 2003 [27]	11 **	Inhalational	No specific cardiac histopathological abnormalities noted
Tabei *et al.* 2004 [33]	33	Cutaneous, Inhalational, and Gastrointestinal	No specific cardiac histopathological abnormalities noted
Babamahmoodi *et al.* 2005 [39]	3	Gastrointestinal	No functional cardiac abnormalities noted
Walsh *et al.* 2007 [35]	1	Inhalational	Echocardiogram showing minimal pericardial effusion
Klempner *et al.* 2010 [37]	1	Gastrointestinal	Transthoracic echocardiogram showed a normal ejection fraction, no valvular vegetations and findings consistent with right atrial volume overload, and right ventricular systolic hypertension
Doganay *et al.* 2010 [40]	22	Cutaneous	No functional cardiac abnormalities noted
Popescu *et al.* 2011 [41]	2	Cutaneous	No functional cardiac abnormalities noted
Powell *et al.* 2011 [42]	1	Injectional	No functional cardiac abnormalities noted
Gruno *et al.* 2012 [43]	3	Injectional	No functional cardiac abnormalities noted
Russel *et al.* 2013 [19]	2	Injectional	Transesophageal echocardiogram reported normal in setting of fulminant septic shock
Booth *et al.* 2014 [2]	27	Injectional	Of nine patients reported, three had dysfunction based on echocardiography, lithium dilution or pulse contour cardiac outputs, and one had an elevated troponin. Other patients had no evidence of abnormal cardiac function (see section 3)
Sprenkle *et al.* 2014 [38]	1	Inhalational	Echocardiogram showed left ventricular hypertrophy with EF of 40%, normal estimated pulmonary artery pressure, probable decreased right ventricular function and no pericardial effusion

* This review included six other patients in this table described as isolated cases including reports by Mayer, Borio, Bush and Mina; ** This review included patients in this table described in case reports by Jernigan and Barakat.

## 4. Effects of LT on the Heart in *in vivo*, *in vitro* and *ex vivo* Studies

Following the identification of LT (and ET) by Smith *et al.* in 1955 and up until the early 1970s, there were multiple studies performed by several groups employing small and large animal models examining LT’s physiologic effects, including its cardiopulmonary ones [45,46]. While some of these studies did examine myocardial histology, and many measured blood pressure and heart rate, there were no studies that employed techniques permitting an investigation of the potential direct myocardial effects of LT. In these investigations, most of which employed a LT challenge administered as an intravenous (i.v.) bolus, the predominant cardiopulmonary changes noted were disruption of the pulmonary arterial endothelium with attendant edema and hemorrhage [47,48]. Hypotension did not occur until very close to the time of death in these models and was attributed primarily to already evident pulmonary dysfunction. Following these early studies, there was limited research examining the cardiovascular effects of LT until the outbreak of infection in the US in 2001. Notable in that outbreak in which patients received aggressive ICU support when needed, and at odds with earlier conclusions regarding LT’s potential cardiovascular effects, was that shock occurred well before death in some non-survivors and was not the result of respiratory failure [1]. However, as described above, shock in patients appeared associated with a particularly poor outcome when compared to other types of bacteria.

Following the 2001 outbreak, there was renewed interest in investigating the cardiovascular effects of LT in both *in vivo* and *in vitro* models. One of the first studies to be published in this regard did not include hemodynamic measures but employed comprehensive histologic examination in combination with biomarker analysis in mice challenged with LT [49]. This study concluded that LT produced non-inflammatory hypoxic tissue injury consistent with a state of hypoperfusion that was tumor necrosis factor α (TNFα) independent. Histology demonstrated minimal myocardial coagulative necrosis with mild cardiac dilation at 48 h in a few animals. However, creatinine phosphokinase measures were notably elevated to high levels in some animals. Our group, employing 24 h LT infusions in rats to better simulate toxin release during infection and measuring blood pressure continuously with indwelling catheters in awake animals, demonstrated that LT produced gradual reductions in blood pressure that were greater in non-survivors than survivors [50]. Lethal toxin did not alter oxygenation or lung or myocardial histology measured on light microscopy. The development of shock with LT was also not associated with the systemic inflammatory response a comparably lethal dose of lipopolysaccharide produced. However, neither of these studies directly examined the effects of LT on myocardial function.

The initial investigations reporting that LT might produce direct myocardial depression were performed by a group employing echocardiography in Sprague-Dawley rats. In one study, animals were challenged with LT as an i.v. bolus [13]. Although the LT dose employed was designed to produce an approximate 50% lethality rate, mean arterial blood pressure (MAP, measured telemetrically) appeared to decrease markedly over a 6 to 8 h period to less than 30 mmHg and subsequent measures were not reported. Echocardiography performed from 0 to 2 h, demonstrated that compared to control animals, LT produced significant increases in both left ventricular systolic and diastolic areas (LVAs and LVAd respectively) and in the velocity of propagation (Vp), a surrogate measure of left ventricular compliance. Left ventricular ejection fractions (LVEF) were reportedly decreased with LT, but calculated values were not provided. It was concluded that LT had induced acute myocardial dysfunction comparable to fulminant myocarditis and manifested by marked increases in ventricular compliance. However, the rapid and marked reductions in blood pressure occurring in the model confound the echocardiographic findings since it is unclear to what degree they contributed to *versus* were the result of the cardiac changes noted. In a subsequent study using a similar LT challenge (i.v. bolus), animals were sacrificed 2 to 5 h after LT and tissue was taken for histological analysis [51]. Light and electron microscopic examination of lung tissue from LT challenged animals demonstrated extensive extravascular fluid and fibrin deposition consistent with pulmonary edema. Unfortunately no analysis was provided of heart tissue and so it is unknown whether there was a histologic basis either on light or electron microscopy for the functional myocardial changes the investigators had previously reported on in this same model. However, examination of liver, trachea, esophagus, kidney, urinary bladder, thyroid, parathyroid, adrenals, pancreas, pituitary, brain, and bone marrow did not show changes. In another *in vivo* study reported by this group, rats had serial blood pressure via non-invasive tail measurements and echocardiography measures at baseline before and from 12 to 48 h after intraperitoneal (i.p.) LT challenge [52]. In this study, LT administration was reported to reduce Vp (as opposed to the increases previously noted), to increase LVAs (but not LVAd) and reduce LVEF. Heart-rate-corrected-velocity of circumferential fiber shortening (VCFC) was also decreased with LT and was reported to reflect a reduction in left ventricular contractility. Based in part on their findings with echocardiography in the rat model, as described next, investigators from this group collaborating with others, did additional *in vivo* and *in vitro* studies to understand how LT might affect heart function at the cardiomyocyte level.

In one series of studies using cardiomyocytes isolated from normal mice and treated with LT or from mice challenged 18 h previously with intraperitoneal (i.p.) LT, the investigators reported that LT inhibited cardiomyocyte peak shortening (PS) and the maximal velocity of shortening and re-lengthening (±dL/dt), and increased the time to peak shortening (TPS) and time to 90% re-lengthening (TR_90_) [15,53,54]. These changes were associated with an inhibitory effect of LT on Ca^2+^ transients within the cardiomyocytes, manifested primarily by a prolongation in Ca^2+^ decay. Experiments with either aponycin, an NADPH oxidase inhibitor or with catalase over-expressing mice, suggested that the inhibitory effects of LT on cardiomyocyte function was in part related to the production of superoxide and reactive oxygen species (ROS). Other experiments suggested that these or additional actions by LT produced cardiomyocyte depression via mechanisms involving; interference with the Ca^2+^ regulatory proteins SERCA2a and phospholamban (PLB); stimulation of autophagy and to a lesser extent endoplasmic reticulum (ER) stress; and the production of mitochondrial membrane or ubiquitin and proteasome defects. However these effects were not always consistent when the methods of cardiomyocyte exposure to LT were compared. For example, while measures 2 h after *in vitro* LT exposure showed up and down regulation of the Ca^2+^ regulatory proteins SERCA2a and phospholamban (PLB) respectively, these proteins were not altered when measured 18 h after *in vivo* i.p. LT exposure. Other experiments in this series of studies in knockout mice suggested that the effects of i.p. LT challenge on cardiomyocyte function were mediated in part by toll-like receptor-4 (TLR-4). Echocardiography in this experiment showed that while LT in wild type mice (WT) reduced left ventricular end diastolic dimensions (LVEDD) and left ventricular end systolic dimensions (LVESD) and calculated fractional shortening and cardiac output, these changes were smaller in TLR-4 knockout animals.

In another study by members of this group, serial echocardiographic measures in Sprague-Dawley rats challenged with a minimally lethal i.v. LT bolus demonstrated very early (2 to 4 h after challenge) evidence of diastolic heart failure with pulmonary artery regurgitation and left atrial dilation, when compared to baseline measurements [16]. Left ventricular ejection fraction was unchanged with LT challenge in this model. Non-challenged animals serving to control for the effects of serial echocardiography and anesthetics (baseline, 2, 4, 8, and 24 h after challenge) were not presented. In data that were compared to a control, LT challenge was noted to increase intracellular Ca^2+^ (Ca^2+^*_i_*), an effect that could result in diastolic dysfunction. Consistent with the known effect of LT on interference with MEK and activation of MAPKs, LT challenge was found to inhibit of MEK7 and JNK1 activity resulting in downstream activation of protein phosphase, dephosphorylation of PLB, and interference with SERCA2a regulated Ca^2+^ uptake by the sarcoplasmic reticulum. The investigators in this study suggested that the early diastolic dysfunction that was observed with LT may have a clinical correlate in the pleural and pericardial effusions and pulmonary edema sometimes noted in patients with *B. anthracis* infection.

While this series of studies is informative, limited data was presented linking the mechanisms speculated to underlie LT’s depressant effects, to the echocardiographic changes (increased or decreased Vp and increased LVAs alone or in combination with increased LVAd) or to the hypotension and lethal effects, LT was originally reported to produce [13,15,52,53,54,55]. While survival was modestly prolonged with an i.p. LT challenge in catalase overexpressing mice compared to wild type animals, these changes were not reported to be significant and no measures of *in vivo* cardiac function were described. Also, in the echocardiography data that was presented in the studies comparing TLR-4 knockout and WT mice, LT was associated with decreases in LVEDD and LVESD, and not the increases (either in both or only the latter) that would have been expected based on the original rat experiments showing increases in LVAd and LVAs [13,52]. Overall, these studies certainly suggest that LT may have important myocardial effects. However they also highlight how the effects of challenge with biological agents in animal models may vary based on the method of challenge and the model employed for study.

Studies by another group have also suggested that direct myocardial depression has a role in LT associated lethality [49]. An initial study noted above in mice challenged with i.p. or i.v. LT, while not showing marked myocardial changes on histology, noted significantly increased creatine phosphokinase levels in animals consistent with myocardial injury. In a subsequent study, this group measured three more selective markers of cardiac injury including myoglobin, cardiac troponin-I (cTnI) and heart type fatty acid binding protein (H-FABP) for up to 120 h after lethal i.p. LT challenge in C57BL/6J mice [14]. They found substantial increases in all three of these markers within the first 15 h after toxin challenge that persisted for up to 48 h. As opposed to light microscopy of heart tissue which showed little change with LT in the model, electron microscopy showed endothelial necrosis, inter-fiber edema with cell debris, altered endothelial junctions, and fragmented myofilaments. Despite these biochemical and ultrastructural changes though, echocardiography of animals 24 h after LT challenge only showed a trend in reduced LVEF. However, the striking and rapid increases in cardiac-selective enzymes with LT in the study suggested that the heart might be an early target of LT in this mouse model. This possibility was explored further in studies employing mice engineered to either lack or to over-express CMG2 in selected tissues [10]. In these elegant studies in mice challenged with i.p. LT, selective deletion of CMG2 in cardiac and vascular smooth muscle tissue together was highly protective and deletion in cardiac tissue alone was still protective but less so. CMG2 deletion in endothelial tissue alone was not protective. Consistent with this, increases in cTnI and decreases in LVEF with LT challenge were reduced or not evident in mice lacking CMG2 expression in cardiac tissue alone or and in cardiac and vascular smooth muscle tissue together. However, at higher LT doses, mice lacking CMG2 in all three cell types were more resistant to LT than cells lacking CMG2 in only cardiac and vascular smooth muscle cells. These results support the possibility that in this mouse model, LT’s effects on cardiac, vascular smooth muscle and endothelial cells all contribute to the toxin’s pathogenic effects, although endothelial cells may only contribute when LT levels are very high. Notably however, while mice lacking CMG2 in all three cell types together were resistant to *B. anthracis* spore challenge, ones lacking it in endothelial cells alone, were not.

Another group studied serial electrocardiograms (ECG), cTnI and echocardiography measures up to 72 h after low or high doses of LT challenge in Dutch belted rabbits [20]. Some animals were also sacrificed at 72 h for histology studies. High but not low LT doses were associated with reductions in MAP first evident at 36 h. Both low and high LT doses were associated with increases in cTnI levels at 48 and 72 h although ECGs were unchanged. Although quantitative measures were not provided, light microscopy studies at 72 h were described as showing mild myodegeneration and subacute inflammation with histiocytes, mononuclear cells, and heterophils present in low dose animals and acute, multifocal cardiac myocyte necrosis with and without mineralization in the ventricles and septum in high dose animals. Despite these increases in cTnI and histologic changes, left ventricular internal diastolic and systolic diameters on echocardiography did not differ from controls in high dose animals at 48 h (when cTnI levels were highest).

These studies of LT’s effects on myocardial function in *in vivo* mouse and rat models, while informative, did not examine this function using methods that controlled for the potential preload and afterload effects of LT. These studies have also not typically measured pulmonary function to determine whether respiratory failure itself might have contributed to myocardial changes with LT. However, a small number of studies have attempted to address these confounding variables.

In a review of their early work, investigators that first reported the effects of LT challenge on echocardiography measures in the rat described data from six canines with indwelling left ventricular pressure-volume loop catheters that were observed for up to 96 h after a lethal dose of LT (four animals) or control (two animals) challenge [55,56,57]. Using these direct measures, the investigators noted that a lethal iv LT challenge was associated with progressive increases in end-diastolic pressures and time of relaxation and a rightward shift of pressure-volume curves with decreases in contractility, left ventricular ejection fraction, and stroke volume, together suggesting the presence of both systolic and diastolic dysfunction. Isolated cardiomyocyte contractile function was determined at the time of the animals’ death or sacrifice and showed a 46% reduction in cell contraction and relaxation in LT challenged animals. This study appears to be a highly informative one but unfortunately its findings have only been published in abstract and review form. Much data such as the course of blood pressure changes occurring in the model would be of considerable value to better assess the myocardial changes described. Also, while this group had reported that LT caused lung injury in the rat, pulmonary findings were not described in this canine report [51]. Therefore, whether pulmonary dysfunction (e.g., hypoxia) contributed to the myocardial changes noted is unclear.

**Figure 1:**
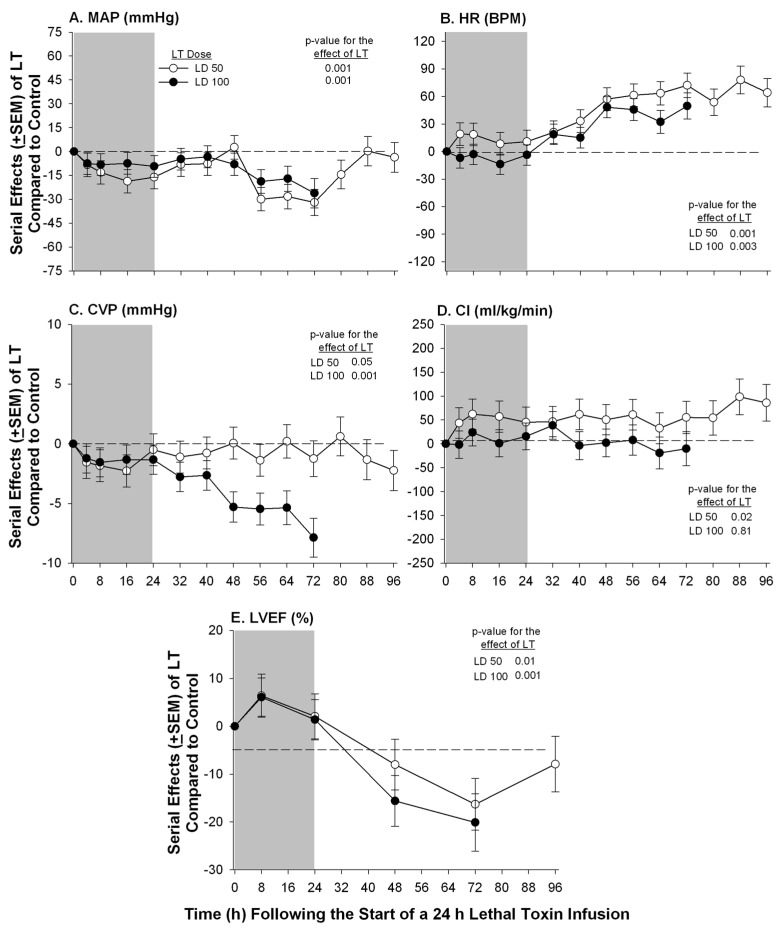
Serial mean effects (± SEMs) of 24 h infusions (shaded areas) of low (LD50) or high (LD100) doses of lethal toxin (LT) compared to protective antigen alone (controls) on changes from baseline in mean arterial pressure (MAP, panel **A**), heart rate (HR, panel **B**), central venous pressure (CVP, panel **C**), cardiac index (CI, panel **D**) and left ventricular ejection fraction (LVEF, panel **E**). The *p*-values shown are for the effects of LT compared to control. Increases or decreases with LT (compared to controls) are indicated by symbols above or below the dashed horizontal no-effect line respectively.

We have examined the effects of LT on myocardial function while attempting to account for its possible preload, afterload, and pulmonary effects, both in canines with indwelling systemic and pulmonary arterial catheters and in isolated rat hearts perfused under constant pressure. In canine studies, sedated and mechanically ventilated animals were challenged with 24 h LT infusions and serial cardiopulmonary measures were obtained for up to 96 h [17]. Compared to controls, both lower LT doses (50% lethal) and higher doses (100% lethal), produced progressive hypotension, increases in heart rate, and reductions in central venous pressure (CVP). Arterial oxygenation did not decrease with either low or high doses of LT throughout the 96 h study period. While neither LT dose altered pulmonary artery occlusion pressure (PAOP), both produced reductions in LVEF first evident 48 h after the initiation of toxin infusion (Figure 1).

Cardiac index did not decrease with either toxin dose possibly because HR increased. On the one hand, decreases in LVEF in the face of unchanged PAOP and the absence of arterial hypoxemia, suggested that LT had potentially depressed myocardial function directly. However, it was also noted that daily normal saline volume loads (40 mL/kg over 40 min) were associated with increased LVEF in animals challenged with high dose LT. This latter finding in combination with the low CVP levels measured with both toxin doses raised the possibility that changes in preload with LT may have contributed in part to the reduced LVEF. In the same LT challenged canine model, compared to no hemodynamic support (fluid and vasopressor therapy titrated to PAOP and MAP respectively) or hemodynamic support alone, hemodynamic support with a PA directed monoclonal antibody increased both CVP, LVEF, MAP, and survival [58].

**Figure 2:**
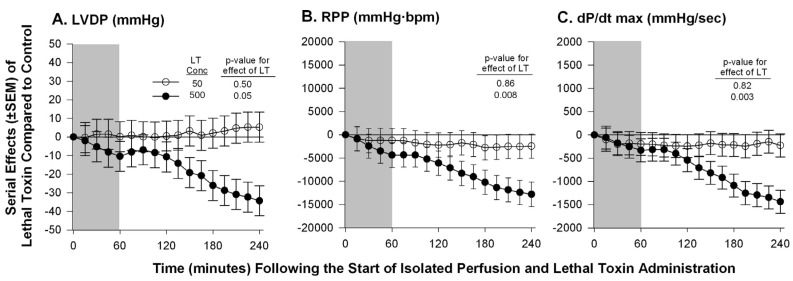
Serial mean effects (± SEMs) of two concentrations of lethal toxin (LT, 50 or 500 ng/mL) compared with protective antigen alone (controls) administered to isolated perfused hearts excised from healthy animals on left ventricular developed pressure (LVDP, panel **A**), rate pressure product (RPP, panel **B**) and rate of change in LV pressure during contraction (dP/dt max, panel **C**). The shaded area represents the time of LT or control administration. The *p*-values shown are for the effect of LT *versus* control. Increases or decreases with LT (compared to controls) are indicated by symbols above or below the dashed horizontal no-effect line respectively. The LT concentration of 50 ng/mL was comparable to a dose previously shown to produce a 50% lethality rate in *in vivo* experiments. Although the concentration of 500 ng/mL did depress myocardial function, this represented a dose 10-fold greater than one producing lethality *in vivo*.

Therefore, to further examine the myocardial effects of LT in a system free of preload and afterload effects, we isolated hearts from healthy Sprague-Dawley rats and measured their function while they were perfused over four hours and under constant pressure in a Langendorff system [59]. In these studies, compared to controls exposure of hearts to a concentration of LT in the perfusion fluid comparable to ones shown to produce shock and lethality in the *in vivo* rat model (50 ug/mL), did not alter any parameter measured including among others: heart rate (HR), left ventricular developed pressure (LVDP), rate pressure product (RPP), dP/dt max, and dP/dt min. Only when the concentration of LT was increased to levels 10-fold (500 ug/mL) greater than ones producing shock *in vivo*, were changes seen including decreases in HR, LVDP and dP/dt max and increases in dP/dt min (Figure 2). The relevance of these latter changes were unclear however since they were evident only with LT concentrations much greater than the doses employed *in vivo*. Furthermore, absence of changes in cardiac function with the lower LT concentration may have been due to inadequate observation time to see changes develop.

**Figure 3:**
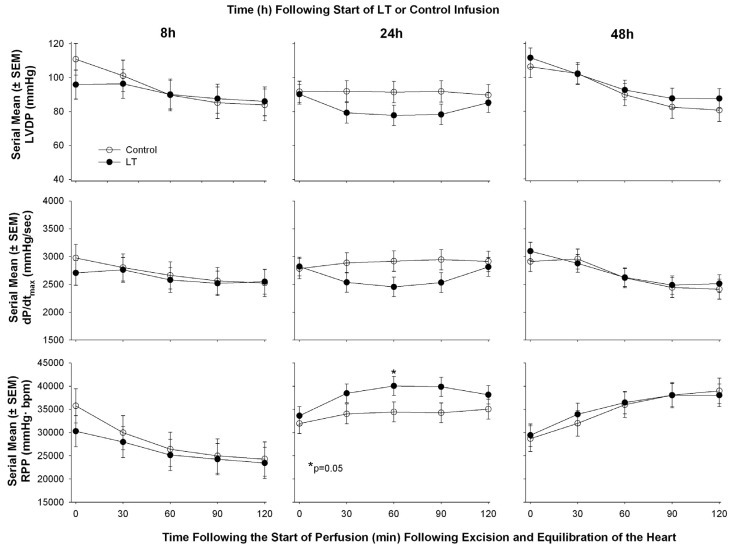
Serial mean (±SEM) left ventricular developed pressure (LVDP), maximum rate of change in LV pressure during contraction (dP/dt max), and rate pressure product (RPP) in hearts excised from animals at either 8, 24, or 48 h after the initiation of an *in vivo* 24 h infusion of lethal toxin (LT) or protective antigen alone (control) and then perfused under constant pressure. The only significant difference (*p* = 0.05) that was noted between LT and control was a decrease in dP/dt max at 60 min of perfusion.

To explore this latter possibility further, we challenged Sprague-Dawley rats with a 24 h infusion of LT in doses that resulted in 30% lethality rates [60]. At 8, 24, or 48 h after the start of LT (or control infusion) animals were randomly selected and had echocardiography performed after which they were sacrificed and their hearts were isolated and perfused under constant pressure in the Langendorff system. On echocardiography, compared to controls, LT challenge decreased left ventricular ejection fraction (LVEF) at 8 and 48 h but increased it at 24 h in patterns that differed significantly over time. Lethal toxin also decreased calculated cardiac output (CO) across the three time points in an overall pattern that was significant. However, once hearts were isolated from animals and investigated independent of potential preload and afterload effects present *in vivo*, prior lethal LT challenge was not associated with significant differences in any parameter, including HR, LVDP, RPP, dP/dt max, or dP/dt min at any time point compared to controls (Figure 3). Table 2 summarizes functional cardiac findings from the pre-clinical studies in lethal toxin challenged models.

**Table 2:** Summary of functional cardiac findings from preclinical studies in lethal toxin challenged models.

Publication	Subjects	Route of Lethal Toxin Exposure	Functional Measurement	Findings
Watson *et al.* 2007 [13]	Sprague-Dawley rat	Intravenous bolus	Echocardiography	▪20% increase in LVAs and LVAd within 2 h. Specific LVEF measurements not reported; ▪Increase in Vp
Watson *et al.* 2007 [52]	Sprague-Dawley rat	Intravenous bolus	Echocardiography	▪30% reduction in LVEF in 11/14 rats surviving after 48 h related to acute increase in LVAs. No increase in LVAd noted; ▪Decreased VCFC, Decrease in Vp
Cheng *et al.* 2007 [57]	Canine	Intravenous bolus	Pressure-Volume catheter	▪Significant LV dysfunction starting at 6 h with development of heart failure at 96 h; ▪Decreases in LVEF, stroke volume, LVESP, contractility, prolonged relaxation time constant, increases in LVEDP
Moayeri *et al.* 2009 [14]	C57BL/6J mouse	Intravenous bolus	Echocardiography	▪Decreases in ejection fraction and fractional shortening at 24 h after LT challenge without change in stroke volume or CO
Sweeney *et al.* 2010 [17]	Purpose–bred Beagle	Continuous infusion	PA Catheter Echocardiography	▪Low and high dose (see section 4) of LT caused progressive declines (15%–20%) in LVEF at 72 h; ▪No significant change in PAOP or SVI with either dose but CVP decreased with high dose
Lawrence *et al.* 2011 [20]	Dutch-belted rabbit	Intravenous bolus	Echocardiography	▪Serial echo measurements at 0 to 48 h showed no significant change in LVAs or LVAd despite elevated markers of myocardial injury
Hicks *et al.* 2011 [59]	Isolated Sprague-Dawley rat heart	Continuous non-recirculating perfusion	*Ex-vivo* Langendorff Model	▪No change in LVDP, RPP, or dP/dt max at a known lethal dose of LT; ▪A 10-fold increase in the lethal dose caused decreases in all measured parameters
Liu *et al.* 2013 [10]	Mouse	Intraperitoneal	Echocardiography	▪Significant decrease in EF at 48 h after LT challenge
Golden *et al.* 2013 [16]	Sprague-Dawley rat	Intravenous bolus	Echocardiography	▪Abnormal indices of diastolic dysfunction within 2–8 h including prolonged LV deceleration time, elevated E/E’ ratio, left atrial chamber enlargement and pulmonary regurgitation; ▪No change in EF noted
Li *et al.* 2015 [60]	Sprague-Dawley rat	Continuous infusion	Echocardiography *in vivo* followed by *ex vivo* Langendorff Model	▪LT decreased CO and decreased LVEF at 8 and 48 h but increased it at 24 h measured with cardiac echo; ▪In isolated hearts following *in vivo* exposure to LT no consistent change at 8, 24, or 48 h in LVSP, LVDP, RPP, or dP/dt max or min

CO: Cardiac output, dP/dt: Rate of change in LV pressure during contraction, LVDP: Left ventricular developed pressure (*LVDP = LVSP − LVEDP*), LVEDP: Left ventricular end diastolic pressure, LVEF: Left ventricular ejection fraction, LVESP: Left ventricular end systolic pressure, LVSP: Left ventricular systolic pressure, LVAs: Left ventricular area in systole, LVAd: Left ventricular area in diastole, PAOP: Pulmonary artery occlusion pressure, RPP: Rate pressure product (*LVDP × HR*), SVI: Stroke volume index, VCFC: velocity of circumferential fiber shortening, Vp: velocity of propagation.

## 5. Conclusions

As recently noted, the mediators and mechanisms underlying hemodynamic instability during *B. anthracis* infection are multifactorial and complex [61]. Likely related to this complexity, findings from clinical reports in combination with *in vivo*, *in vitro* and *ex vivo* studies provide a mixed picture as to whether *B. anthracis* infection is associated with myocardial depression or whether LT itself can cause this depression directly and independent of its other systemic effects. Interestingly, in the one clinical report providing both echocardiography and pulmonary arterial catheter data in a patient requiring vasopressor therapy and who ultimately died, left ventricular dysfunction other than that related to a progressive pericardial effusion appeared minimal [18]. While data from echocardiography studies in other patients are more limited, several reports from patients with severe disease have also not been remarkable [19,37]. In those reports where dysfunction has been noted, whether this was related to prior disease in older patients or to infection itself is unclear since follow-up studies after resolution of infection were not provided. Comparison of animal studies with LT alone may be difficult to interpret based on the multiple factors that differed between them (e.g., differing species and models employed and methods of toxin administration) as well as the fact that many of these studies did not account for changes in preload and afterload with LT or with changes in pulmonary function. However, even in models accounting for such influence, the effects of LT on cardiac function have been variable.

In conclusion, if LT does contribute to shock during anthrax infection, presently available clinical data do not provide strong evidence that this compromise is related to direct myocardial depression. However, efforts should continue to be made to further understand the impact of LT on myocardial function in anthrax sepsis both clinically and in preclinical models. This question carries important implications not only for the conventional management of patients with anthrax infection and shock but also for the development of new and targeted therapies.
